# Telemedicine using an iPad in the spinal cord injury population: a utility and patient satisfaction study

**DOI:** 10.1038/s41394-018-0105-4

**Published:** 2018-08-08

**Authors:** Samantha Sechrist, Sarah Lavoie, Cria-May Khong, Benjamin Dirlikov, Kazuko Shem

**Affiliations:** 1 Rehabilitation Research Center, Santa Clara Valley Medical Center, San Jose, CA United States; 2 Department of Physical Medicine and Rehabilitation, Santa Clara Valley Medical Center, San Jose, CA United States

## Abstract

**Study design:**

Prospective observational.

**Objectives:**

To explore participants’ experience, satisfaction, and utility of telemedicine.

**Setting:**

Spinal cord injury (SCI) rehabilitation clinic at a county hospital.

**Methods:**

Participants in this study received telemedicine appointments for routine scheduled care and/or urgent consults with a spinal cord injury specialist via iPad on FaceTime. Demographic changes, health care utilization, and medical complications were assessed. A Program Satisfaction Survey (PSS) was completed after a 6-month enrollment.

**Results:**

Telemedicine visits included general follow-ups (51.25%), “multiple issues” (24.38%), skin (6.88%), bowel and bladder (5.63%), spasms (3.13%), and pain (3.13%). The PSS was collected (*n* = 45) and revealed positive results in perceived health, satisfaction with equipment/ease of use, and satisfaction with the program. Analysis of anecdotal comments revealed themes such as efficiency, convenience, and reduced barriers provided by telemedicine visits.

**Conclusions:**

This study shows the feasibility and acceptance of a telemedicine intervention via iPad for individuals with SCI through positive PSS ratings and the wide variety of clinical topics addressed.

**Sponsorship:**

Craig H. Neilsen Foundation.

## Introduction

The impact of spinal cord injury (SCI) on the lives of affected individuals is far-reaching. Subsequent paralysis, limited mobility, and secondary complications, such as urinary tract infections, pressure ulcers, mental disorders, musculoskeletal, digestive, respiratory, and circulatory diseases, present many unique challenges following discharge from acute inpatient rehabilitation [1]. The medical complexities associated with SCI frequently result in re-hospitalizations [1–3] as well as the need for ongoing medical and psychological care [4]. Thus, it is critical to provide individuals with SCI with accessible specialized long-term care.

Vulnerable populations, such as individuals with disabilities and chronic conditions including SCI [5, 6], are disproportionally disadvantaged when accessing health care services [7, 8]. Continuation of care can also be impacted by geographical, physical, and transportation barriers, especially among individuals living in rural areas that may need to travel long distances to receive immediate medical care or to be seen by an SCI specialist [9]. Consequently, diagnosis and treatment care plans for secondary complications may be delayed, and quality of life (QoL) for individuals with SCI can be hindered [10]. Telemedicine (TM) can improve QoL by addressing a wide variety of clinical issues without the person with SCI needing to leave home or rely on a caregiver. Reducing psychological stress caused by mood disorders and anxiety over accessing specialized care, efficiently providing high-quality information to stay healthy, and addressing lifestyle changes that allow persons with SCI to remain engaged in the community are also potential benefits of TM for persons with SCI [4, 11].

TM utilizes technology and mobile devices such as smartphones, tablets, and laptops to deliver health care services and increases the accessibility of health information and services to users [12–14]. Utilizing and integrating TM into clinical practices may offer a low-cost alternative to traditional in-person medical care [15] by providing access to timely care from an SCI specialist regardless of physical or geographical limitations. This is of particular importance for individuals that reside in rural areas with limited access to medical services [9].

TM studies among individuals with SCI have focused on the prevention, management, and treatment of pressure sores [16–18], chronic pain [19, 20], and telepsychology interventions for mental health issues [21] including depression [22]. The majority of previous TM research, among the SCI population, has been conducted via telephone [17, 18, 21–25], videoconferencing [13, 15, 17, 26], or web-based portal platforms [27].

To our knowledge, TM programs that use iPads to facilitate participant–provider communication via the FaceTime videoconferencing application, specifically, has not been explored. The iPad was selected as the TM device of choice given its established ease of use for videoconferencing, portability, familiarity, and ease of use for participants with physical limitations including impaired hand function. For example, the touch screen allows for easier use than a device with push-buttons, and the iPad has adaptive equipment available such as wheelchair mounts and mouthsticks. Additionally, for this study, the iPad FaceTime via the Verizon data plan ensured double-encryption for security and the platform was approved by the hospital’s Information Services Department for TM purposes. Research elucidating participant satisfaction of TM programs is not well-established, as few studies have highlighted participant experience and satisfaction with TM [28, 29]. Therefore, the objective of this study was to explore participants’ experience and satisfaction with TM as well as ease of use with TM technology and adaptive equipment.

## Methods

### Setting

This is a single-center prospective observational study conducted at a rehabilitation clinic at a county hospital in California, USA. This study was carried out with the approval of the Institutional Review Board’s Research and Human Subjects Review Committee of Santa Clara Valley Medical Center.

### Participants

Sixty-two participants (48 males, 14 females) (Table 1) were recruited from an acute inpatient rehabilitation program and outpatient clinic. Participants were 18 years or older at the time of enrollment and had a traumatic or non-traumatic SCI at any neurological level. Participants were excluded who were unable to communicate in English as the health care providers were strictly English-speaking, lived outside the state of California, and/or had insurance known to not approve TM visits with the study provider. Participants were recruited for the study from within the state of California and outside of Santa Clara County. Many of the participants were recruited from Northern California/Oregon border, Central California, and Southern California, specifically residing in communities lacking specialty SCI care. All participants included in the study completed and signed the informed consent forms and the Health Insurance Portability and Accountability waiver.

**Table 1:** Patient characteristics (*n* = 62)

Characteristics	Mean (std)
Age at enrollment	41.3 (16.1)
Sex	*n*
Male	48
Female	14
Ethnicity	*n*
Caucasian	34
Hispanic	14
Asian	8
African American	3
Other	3
Education	*n*
Less than high school	7
High school/GED	16
Trade	4
Some college	21
Bachelors	9
Masters or PhD	3
Other	1
Unknown	1
Etiology	*n*
Motor vehicle accident	21
Other	19
Fall	15
Gunshot wound	4
Sports	3
Level of injury	*n*
Cervical	41
Thoracic	21
AIS	*n*
A (complete)	30
B, C, D (incomplete)	32

Table represents the demographic characteristics, frequency, and mean of enrolled participants

*std* standard deviation, *n* count

### SCiPad program description

This program provides individuals with an SCI a live interactive TM consultation with a board-certified SCI specialist. The TM consultation was completed through Apple’s FaceTime via an iPad. All participants received an Apple iPad Air and a 6-month long cellular data plan. Training on how to use an iPad and the FaceTime application was provided by the program coordinator. Occupational therapists (OT) were consulted to assess the need for assistive technology and to provide training, if necessary. Participants received a hand stylus, iPad case/stand, as well as a mouthstick and wheelchair mount, if indicated by an OT. Participants were also given blood pressure (BP) cuffs to manage BP at home.

Participants contacted the program coordinator to schedule a TM appointment when seeking advice from the physician regarding a condition related to their SCI. The program coordinator acted as the liaison between participant and physician and was available to participants between 9 AM and 4 PM on weekdays for non-emergency needs. TM encounters with an SCI specialist could be set up within 24 h on weekdays during office hours, if necessary. BP values were reported to the physician during TM visits to monitor any pertinent changes. Using the audio visual capability of FaceTime, the study physician could hear the participant’s voice (e.g., strong or weak), and could also visualize the participant’s physical condition, such as mood, skin color, rash, ability to move arms and legs, presence of spasms, and ability of the participant to move in bed or in a wheelchair. Pressure ulcer(s) could also be visualized, usually using assistance from a caregiver. Any necessary laboratory orders, such as urinalysis, were written by the physician and faxed to the participant’s local laboratories. Subsequently, lab results were faxed back to the physician’s office for review.

Participants were followed for 6 months and were contacted monthly by the program coordinator to complete follow-up interviews that assessed demographic updates, health care utilization, and medical complications. At baseline and during the 6-month follow-up, participants also completed questionnaires focused on QoL and psychosocial outcomes.

### Measures

Upon conclusion of the study, participants were asked to complete a 20-question Program Satisfaction Survey (PSS). Questions ranged from individuals’ experience with the iPad device, the overall program, preferences for medical care, and anecdotal comments. Part A consisted of 13 questions on a Likert-type scale (1: strongly agree to 7: strongly disagree; 8: not applicable); 11 of these questions were of a positive tone and two were of a negative tone. Part B consisted of five multiple choice questions regarding the frequency of iPad use, purpose for iPad use, others’ use of the iPad, cellular connectivity, and medical care preference. The last two questions were free response for collection of anecdotal comments regarding reasons why a participant did not have TM visits during the 6-month duration, as well as general comments or suggestions.

### Data analysis

Demographic information was analyzed to provide a description of participant characteristics. Data regarding TM visits conducted with all study completers were assessed to capture information regarding total number of TM appointments conducted, average length of TM visits, and topics covered during TM encounters.

For analysis of the TM PSS, Part A was divided into three categories: Perceived Health (PH), Satisfaction with Equipment/Ease of Use (SE/EoU), and Satisfaction with Program/TM (SP/TM). The responses for the two negative-toned questions were inverted, and the modes for each theme were calculated to highlight the most common response. Overall, responses for the first 18 questions (Parts A and B) were totaled and percentages were calculated for a full description. The two free response questions were analyzed to reveal themes in participants’ comments.

## Results

### Participant characteristics

In total, there were 62 participants who were enrolled (53 from inpatient rehabilitation and nine from outpatient clinic). Participant characteristics including sex, average age, ethnicity, education level, cause of injury, and level of injury are provided in Table 1. Overall, five participants did not complete the 6-month study (two expired, two were lost to follow-up, and one dropped out) for an attrition rate of 8.65%. The cause of death for the two expired patients was cancer and unknown; however, persons with SCI are also known to have a significantly higher mortality rate in the first year of injury [1].

### TM encounters

**Fig. 1:**
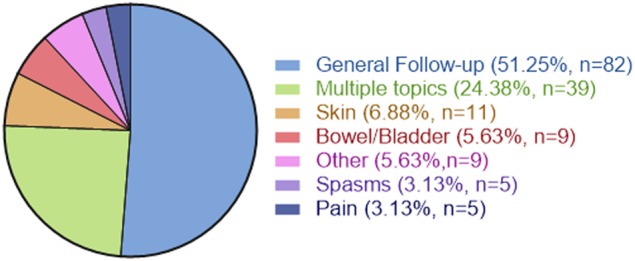
Telemedicine consultation topics represents the percent of telemedicine consultation topics supported by this program

A total of 161 TM visits occurred via FaceTime including six phone calls for advice given by physicians covering in the absence of the principal physician. The average length of TM visits was 23 min (range 5–60 min). The physician was able to conduct TM visits covering topics such as general comprehensive follow-ups, “multiple issues”, skin, bowel and bladder, “other issues”, spasms, and pain (Fig. 1). “Other” issues included fever, sexuality, reviewing of laboratory results, equipment, surgery, BP, and dry mouth.

TM visits in which participants had a need to discuss “multiple issues” were further analyzed to capture topics covered within those 39 visits; a total of 98 issues were found and categorized into 11 different topics: 26.53% (*n* = 26) discussed bowel and bladder, 17.35% (*n* = 17) pain, 16.33% (*n* = 16) spasms and stiffness, 14.29% (*n* = 14) “other” issues, 6.12% (*n* = 6) skin, 4.08% (*n* = 4) pulmonary, 3.06% (*n* = 3) equipment/wheelchair, 3.06% (*n* = 3) BP, 3.06% (*n* = 3) medication/prescription, 3.06% (*n* = 3) therapy/exercise, and 3.06% (*n* = 3) discussed mood. “Other” issues in the multiple issues category included tone, upper extremity movement, hospitalization, poor sleep, headaches, autonomic dysreflexia, insurance problems, weakness, ingrown toenails, edema, and hematoma.

### Program satisfaction survey

Of the 57 program completers, 45 completed the PSS at the end of the 6-month period (78.95% response rate).

Part A of the survey was organized into three categories for analysis (PH, SE/EoU, and SP/TM). Due to the non-normal distribution of survey results, the mode for each category was abstracted. The modes for PH, SE/EoU, and SP/TM were two, one, and one, respectively (1: strongly agree, 2: agree), therefore satisfaction with these three aspects of the program was high.

Full results for Parts A and B of the PSS are shown in Table 2. The results show that 100% of responders ranged from slightly agree to strongly agree recommending the TM program and 88.90% (*n* = 40) believed the care received through TM was just as good as seeing a physician or nurse in person. Additionally, responders believed that the TM program staff responded to needs sufficiently. The responses for top preference for medical care were mixed with 55.56% (*n* = 25) preferring TM, 35.56% (*n* = 16) preferring in-person physician appointments, and 6.67% (*n* = 3) preferring telephone contacts.

**Table 2:** SCiPad Program Satisfaction Survey (*n* = 45)

Part A	Strongly agree	Agree	Slightly agree	Neither	Slightly disagree	Disagree	Strongly disagree	NA
*Perceived health*								
Since receiving the iPad, I have been motivated to monitor my health.	12 (26.7)	17 (37.8)	7 (15.6)	6 (13.3)	1 (2.2)		2 (4.4)	
I feel my health has improved because of the telemedicine program.	15 (33.3)	18 (40)	4 (8.9)	7 (15.6)	1 (2.2)			
*Satisfaction with equipment/Ease of use*								
The training I received helped me to understand how to operate my iPad.	25 (55.6)	10 (22.2)		5 (11.1)			1 (2.2)	4 (8.9)
The iPad was easy to use.	27 (60)	14 (31.1)	3 (6.7)	1 (2.2)				
I was satisfied with the quality of the visual image and audio sound during my TM visit(s).	25 (55.6)	12 (26.7)	2 (4.4)	2 (4.4)			1 (2.2)	3 (6.7)
The iPad took too much time to use.				1 (2.2)	1 (2.2)	13 (28.9)	30 (66.7)	
I was worried about my privacy with the iPad.	2 (4.4)		1 (2.2)	2 (4.4)		16 (35.6)	23 (51.1)	1 (2.2)
I am satisfied with my use of the iPad.	28 (62.2)	15 (33.3)	2 (4.4)					
The adaptive equipment I received was sufficient for my needs.	23 (51.1)	10 (22.2)	4 (8.9)	1 (2.2)		2 (4.4)		5 (11.1)
*Program/Telemedicine satisfaction*								
The care I received through telemedicine was just as good as seeing my physician or nurse.	21 (46.7)	16 (35.6)	3 (6.7)	1 (2.2)	1 (2.2)	1 (2.2)		2 (4.4)
I would recommend the telemedicine program.	33 (73.3)	11 (24.4)	1 (2.2)					
I would like to continue to have telemedicine visits with my physician or nurse.	25 (55.6)	10 (22.2)	3 (6.7)	3 (6.7)		1 (2.2)	1 (2.2)	2 (4.4)
Staff responded to my needs sufficiently.	35 (77.8)	9 (20)	1 (2.2)					

**Part B**	Daily	Multiple/wk	Once/wk	<Once/wk	Other	NA		
How frequently did you use your iPad?	25 (56.6)	15 (33.3)	3 (6.7)		1 (2.2)	1 (2.2)		
	Videos	Games	Internet	E-mail	TM	Music	Other	NA
What purpose did you use your iPad for? (Most frequent purpose shown here only)	4 (8.9)	5 (11.1)	21 (46.7)	4 (8.9)	5 (11.1)	1 (2.2)	4 (8.9)	1 (2.2)
	Family	Caregiver	Friend	Other	None	NA		
Did someone other than you use the iPad?	18 (40)	2 (4.4)		1 (2.2)	23 (51.1)	1 (2.2)		
	Yes	No	NA					
Do you have WiFi at home?	41 (91.1)	3 (6.7)	1 (2.2)					
	TM	In-person	Phone	NA				
Top preference for medical care	25 (55.6)	16 (35.6)	3 (6.7)	1 (2.2)				

Table represents the questions asked in the Program Satisfaction Survey and corresponding responses. Values are frequencies and (percentages)

*NA* not applicable, *TM* telemedicine, *wk* week

Six of the survey respondents did not utilize TM during the 6-month study. From PSS comments, two participants had issues with insurance authorization, one had internet access issues, and one saw the principal physician in person due to coordinating other in-person appointments at the study site facility on the same days.

Analysis of the general anecdotal comments revealed three predominant themes (improved access to SCI specialists, satisfaction with iPad technology/equipment, and general appreciation) with subthemes including quality of care, reduced barriers, improved social support, access to information, advanced medical care practices, efficiency and convenience, and experience with staff.

## Discussion

There is a limited but growing body of research suggesting the benefits of TM for individuals with SCI across multiple domains. Dorstyn et al. reported that telephone-based psychological counseling improved depression, anxiety, and coping skills as compared to standard of care in individual with acquired physical disability, including SCI [30]. A 2001 report compared standard of care to telephone-based or video-based educational interventions with a study nurse aimed to reduce secondary complications after SCI. This study found that the intervention groups had reduced mean annual hospital days and improved QoL 1 year post-discharge [31]. Positive results have also been observed in videoconferencing-based interventions as well. Videoconferencing was an effective method for implementing and monitoring a home exercise program targeting pain reduction and increased shoulder function [32]. In another study, improvement in dental hygiene behaviors were shown at 6 and 12 months following 3 months of dental hygiene training provided by OTs via videoconferencing. Although the authors could not attribute specific improvements to TM or adaptive devices (e.g., power toothbrush), this study further illustrates the variety of applications for TM [33].

Results from our study suggest that TM via iPad can be used to address a variety of issues. Most participants utilized TM for scheduled general follow-up assessments or made an appointment to discuss multiple issues that they were experiencing. A further analysis of TM appointments revealed that the top issues reported matched common complications experienced by persons with SCI in the first year of injury, such as skin (e.g., pressure sores), bowel/bladder (e.g., urinary tract infections), spasticity, and pain [34]. This program provided an easy way to connect participants with a provider to supplement outpatient appointments and ongoing post-rehabilitation guidance for management of care.

The results from the PSS demonstrate the ease of use and general acceptance of TM via iPad specifically as a tool for receiving specialized SCI medical care. The physician was able to address many issues, providing evidence for the wide utility this program can support. Survey results were positive overall, suggesting a broad range of treatable symptoms and satisfaction with the treatment. Results also demonstrate that SCI patients may use TM technology with the appropriate adaptive equipment. These results are in line with studies demonstrating positive patient experiences using TM [28, 29]. It is necessary to explore the integration of TM equipment for populations with functional limitations, such as SCI [11]. Despite growing evidence to support the clinical utility and versatility of TM via mobile technology, literature surrounding the delivery of care through TM for the SCI population has been limited [11, 35].

Anecdotal comments/feedback also support the use of TM. General themes included high-quality care, reduced barriers to accessing SCI specialists, adoption of iPad technology for medical care, efficiency of care received, and positive experiences with program staff. Results indicate that participants were able to connect and discuss medical issues with an SCI specialist that would have otherwise required cumbersome travel to a specialty physician’s office. The data safety considerations of this intervention (e.g., double-encrypted data, password protection, data storage on electronic medical record only) provides confidence that the iPad can be a potential device that other health systems can safely use for TM. The iPad is desirable because it is a multi-purpose device for participants (i.e., participants can use the iPad for many other activities other than TM), it is a well-known device that most people may already own, it is portable, and it is easy to use for participants who have physical limitations such as impaired hand function.

It is also important to note that the survey shows that most of the participants have access to a Wi-Fi connection at home. Even after the 6-month study was completed, participants were given the option to continue seeing an SCI specialist through FaceTime. If participants have a Wi-Fi connection at home, participants in our study could continue their TM visits without the cost of a cellular data plan.

### Limitations

Potential bias may exist in our study in that not all participants completed the survey. Therefore, it cannot be determined whether or not non-responders’ answers would have affected the current results or whether these results are greater than standard of care. Another limitation in this study is that this program did not require participants to have a certain number of TM visits. Future studies may consider assessing the efficacy of TM in a randomized study with a control group; however, it is important to note that this program and the participant feedback reflect the reality of how patients would likely use this service since there were no limits to TM use. Additionally, the exclusion of non-English speaking participants in the study may have led to a loss of potential important data in this population.

## Conclusions

TM increases access to and options for SCI care and reduces physical, transportation, and resource barriers that are frequently experienced by the SCI population. More research evidence for TM in SCI may be needed to justify insurance coverage and implementation of TM into standard of care. Based on comments from the participants who did not have any FaceTime appointments, lack of insurance authorization was one common obstacle faced that prevented them from contacting the physician. Despite study weaknesses, this is the first published project using the iPad to provide TM in the SCI population and to show its utility and acceptance of this intervention/equipment through positive feedback from participants. Proving the acceptance and effectiveness of TM in the SCI population will provide further evidence to support and implement TM programs for persons with spinal cord injuries and disorders.
